# Deficiency of *TOP1MT* enhances glycolysis through the stimulation of PDK4 expression in gastric cancer

**DOI:** 10.1186/s40170-024-00330-w

**Published:** 2024-01-10

**Authors:** Hongqiang Wang, Xutao Sun, Chen Yang, Ziqi Li, Danwen Jin, Wenwen Zhu, Ze Yu

**Affiliations:** 1 Cancer Chemotherapy Center, Zhoushan Hospital, Wenzhou Medical University, Zhoushan, China; 2 Department of General Surgery, Zhoushan Hospital, Wenzhou Medical University, No. 739 Dingshen Road, Lincheng New District, Zhoushan, Zhejiang 316021 China; 3 The Laboratory of Cytobiology and Molecular Biology, Zhoushan Hospital, Wenzhou Medical University, Zhoushan, China; 4 Department of Pathology, Zhoushan Hospital, Wenzhou Medical University, Zhoushan, China

**Keywords:** Gastric cancer, TOP1MT, PDK4, Glycolysis, Cell migration

## Abstract

**Background:**

Abnormal glucose metabolism is one of the determinants of maintaining malignant characteristics of cancer. Targeting cancer metabolism is regarded as a new strategy for cancer treatment. Our previous studies have found that TOP1MT is a crucial gene that inhibits glycolysis and cell metastasis of gastric cancer (GC) cells, but the mechanism of its regulation of glycolysis remains unclear.

**Methods:**

Transcriptome sequencing data, clinic-pathologic features of GC from a variety of public databases, and WGCNA were used to identify novel targets of TOP1MT. Immunohistochemical results of 250 patients with GC were used to analyze the relative expression relationship between TOP1MT and PDK4. The function of TOP1MT was investigated by migration assays and sea-horse analysis in vitro.

**Results:**

We discovered a mitochondrial topoisomerase I, TOP1MT, which correlated with a higher risk of metastasis. Functional experiments revealed that TOP1MT deficiency promotes cell migration and glycolysis through increasing PDK4 expression. Additionally, the stimulating effect of TOP1MT on glycolysis may be effectively reversed by PDK4 inhibitor M77976.

**Conclusions:**

In brief, our work demonstrated the critical function of TOP1MT in the regulation of glycolysis by PDK4 in gastric cancer. Inhibiting glycolysis and limiting tumor metastasis in GC may be accomplished by suppressing PDK4.

**Supplementary Information:**

The online version contains supplementary material available at 10.1186/s40170-024-00330-w.

## Introduction

Worldwide, GC is one of the most common cancers, with more than one million new cases each year, accounting for 5.7% of all cancer diagnoses. The incidence of GC varies greatly by region, with a particularly high incidence in East Asia and Eastern Europe [1]. It is alarming to note that early-onset gastric cancer, which is less than 50 years old, is on the rise in some countries [2]. Early-onset gastric cancer may be related to autoimmune gastritis, intestinal microbial disorders caused by the abuse of antibiotics and acid suppressors, as well as genetic influences. Overall, advanced gastric cancer (including gastroesophageal junction cancer) is still one of the cancers with the worst prognosis, and the 5-year survival rate of patients is only about 6% [3, 4]. It is urgent to improve the survival of patients through effective systematic drug therapy, especially precise targeted therapy.

The Warburg effect, characterized by the utilization of the glycolytic pathway by cancer cells to produce energy even in the presence of oxygen, is a hallmark of GC. Numerous studies have established a correlation between active aerobic glycolysis and the onset and progression of gastric cancer, with several molecular mechanisms being implicated [5]. In a prior investigation, glucose uptake in patients with stomach cancer was assessed by F-fluorodeoxyglucose (FDG)-PET, and all gastric malignancies with high levels of Glut-1 showed improved glucose uptake, with the exception of signet-ring cell carcinoma [6]. In addition, DNA polymerase is also thought to be involved in maintaining mitochondrial homeostasis. It has low expression in GC cells and can bind to PKM2 to limit glycolysis. By increasing the activity and expression of GLUT1, HK2, LDH, and PDK, the metastatic-associated in MACC1 protein can enhance the Warburg effect [7].

The Warburg effect can be inhibited to have direct or indirect therapeutic benefits by slowing the proliferation of GC cells and lowering the amount of medication resistance [8]. For instance, Citrate, a PFK inhibitor, significantly reduced the development of BGC-823 and SGC-7901 GC lines while promoting the mitochondrial death pathway [9].

An important mitochondrial DNA topoisomerase, mitochondrial topoisomerase 1 (TOP1MT), is made up of 601 amino acid peptides. Nuclear localization signals are absent from the N-terminal region, which has mitochondrial localization sequences. As a result, the TOP1MT protein is restricted to mitochondria, where it supports cellular glucose metabolism and preserves the expression of mitochondrial DNA [10–13]. According to reports, TOP1MT sustains cancer cell proliferation in a metabolically unfavorable environment, which enhances tumor size and frequency in animal models. This study’s findings highlight the significance of mitochondria for the proliferation of cancer cells, which moderates the Warburg effect hypothesis.

In our early studies, we found that TOP1MT loss boosted tumor metastasis in GC cells by encouraging GC cell invasion and migration, increasing glucose consumption, lactate generation, and LDHA activity in vitro and in vivo [14]. Therefore, our current work is a continuity study. Interestingly, using bioinformatics and in vitro tests on GC cells, we discovered that TOP1MT can regulate pyruvate dehydrogenase kinase 4 (PDK4), a crucial glycolysis and TCA cycle conversion factor, in addition to LDHA. Reduced TOP1MT can increase PDK4 expression and encourage GC cells to utilize glycolysis.

## Materials and methods

### Data collection

We obtained RNA-seq data for GC and its corresponding clinical information from the TCGA repository. The candidate datasets were selected based on specific criteria, including human gene expression profile, GC specimen, patient gender, availability of follow-up time information (overall survival), and pathological grade. Sixty-two genes involved in the metabolic pathways of gluconeogenesis and glycolysis were obtained from the KEGG_GLYCOLYSIS.v2023 gene set in the Molecular Signatures Database of GSEA.

### Identification of differentially expressed genes (DGEs)

To investigate the influence of glycolysis typing on gastric cancer, we utilized the LIMMA R package, which employs linear models for microarray data, to identify differentially expressed genes (DEGs) between cluster 1 and cluster 2. The cutoff criteria were set at a *P* value < 0.05 and an absolute log2 fold change > 1.

### STAD subclass identification based on glycolysis-related genes

A total of 62 genes associated with glycolysis were used for NMF clustering. The objective of NMF was to identify potential attributes in gene expression patterns through the decomposition of the original matrix into multiple non-negative matrices. Unsupervised NMF clustering was conducted on the metadata set using the R package ‘NMF’. The analysis involved 1000 repeat samples and a maximum grouping limit of 6. The optimal *k* value was determined through the use of the cumulative distribution function (CDF) and consensus heat map. Subsequently, the TCGA-STAD samples were categorized into distinct clusters based on the glycolysis level of tumor tissues.

### Prognosis analysis

We used the Kaplan–Meier technique to plot survival curves correlating the expression levels of four genes (TOP1mt, PGM5, PDK4, and CYC1) with the overall survival time (OS) of STAD patients. The significance of the distinction was assessed using the log-rank test, and genes with a *P* value < 0.05 were chosen for further examination.

### Patients and tissue samples

The Zhoushan Hospital Ethics Review Board approved our research. We obtained paraffin-embedded tissue samples from 250 patients diagnosed with GC at our hospital from 2006 to 2011. These patients did not undergo neoadjuvant chemotherapy before surgery.

### Immunohistochemistry

The immunohistochemical staining followed a previously published protocol [15]. Staining intensity was assessed using a scale ranging from 0 (no staining) to 3 (intense staining). The staining extent was scored based on the percentage of positively stained cells, ranging from 0 (score 0) to 100% (score 4), with intermediate scores of 1 (1–25%), 2 (26–50%), and 3 (51–75%). The staining scores for TOP1MT and PDK4 expression were determined by multiplying the intensity and extent scores, yielding a range from 0 to 12. Tumor tissues were categorized based on their final staining scores as either low TOP1MT expression (scores < 6) or high TOP1MT expression (scores ≥ 6).

### Western blotting

Initially, the protein concentration of each sample was determined, and the sample amount was controlled at 30ug/well according to the protein concentration and the sample volume, and then, proteins were isolated using 10% SDS-PAGE (Beyotime, China) and transferred onto 0.45 μm PVDF membranes (Millipore, USA). After the addition of 5% BSA, the membranes were incubated overnight at 4℃ with the following antibodies: anti-TOP1mt (Proteintech, 16,540–1-AP, China), anti-PDK4 (12,949–1-AP), and anti-β-actin (20,536–1-AP). The membranes were incubated with a secondary antibody conjugated with horseradish peroxidase at 4℃ overnight. The signal was detected using enhanced chemiluminescence.

### Cell culture and transfection

Two GC cell lines, HGC-27 and MGC-803, were purchased from the Chinese Academy of Sciences Cell Bank (Shanghai, China). The cell lines were cultured in a chamber with 5% CO_2_, at a temperature of 37℃, and in a humid environment. The cells were cultured in RPMI-1640 medium supplemented with 10% fetal bovine serum (FBS). The TOP1MT-siRNA mimics and negative control (NC) were procured from Tsingke (Beijing, China). A total of 150,000 cells were transfected with siRNA mimics using Lipofectamine™ 3000 transfection reagent (Thermo Fisher, USA) in 6-well plates, following the manufacturer’s guidelines. After a 48-h transfection period, the cells were collected and used for further experiments. The Supplementary material includes a list of siRNA sequences targeting TOP1MT. M77976 [16], a PDK4 inhibitor, was obtained from MCE (No. 394237–61-7).

### Cell migration

The upper chamber was seeded with cells (1 × 10^4^) possessing a membrane with 8.0 μm pores (Corning, USA). The lower chamber was supplemented with a culture medium containing 10% fetal bovine serum (FBS). After incubating at 37℃ for 48 h, cells in the upper compartment were fixed with paraformaldehyde for 15 min and treated with crystal violet (0.1%) for 10 min. After three rounds of rinsing with double-distilled water, the chamber was stained with a 0.1% crystal violet solution. The number of migrating cells was counted using an inverted microscope after they were dried.

### PH staining

BCECF AM is a fluorescent dye that can penetrate cellular membranes [17]. BCECF AM does not exhibit fluorescence and is converted into BCECF by intracellular esterase after entering the cell, leading to its accumulation within the cell. BCECF can be excited to form green fluorescence at the right pH value. The treated cells were stained with BCECF AM probes according to the provided guidelines. Finally, the cells were viewed using an inverted fluorescence microscope and photographed.

### Measurements of extracellular acidification and oxygen consumption rate

The Seahorse Bioscience XF96 Extracellular Flux Analyzer was employed to monitor real-time changes in cellular respiration and glycolysis rate, as described in reference [18]. The XF96 Extracellular Flux Analyzer from Seahorse Bioscience was used to detect rapid and real-time changes in cellular respiration and glycolysis. In summary, a cell population ranging from 5000 to 20,000 cells per well was cultured in XF96 microplates. The extracellular acidification rate (ECAR) is used as an indirect indicator of glycolysis rate by lactate excretion, while cellular respiration is determined by oxygen consumption (OCR). All measurements were conducted following the manufacturer’s guidelines and procedures.

### Statistical analysis

The data analysis was performed using the R programming language. The Wilcoxon and Kruskal–Wallis tests were conducted to analyze continuous variables that exhibited non-normal distribution. The study employed chi-square analysis to examine categorical variables. Univariate and multivariate Cox regression analyses were conducted to determine if the variable acted as an independent prognostic factor. The Kaplan–Meier method and log-rank test were employed to estimate survival curves and identify any significant differences between them. A *p* value below 0.05 (*p* < 0.05) is deemed statistically significant according to two-sided statistical tests.

Some of the bioinformatic analysis methods (*construction of risk score model by multivariate Cox, construction and validation of a predictive nomogram, DCA (decision curve analysis), mutation of TOP1MT in gastric cancer, and WGCNA analysis*) and mass spectrometry are shown in the Supplementary materials.

## Results

### Identification of glycolysis-related genes in TCGA-STAD

**Fig. 1:**
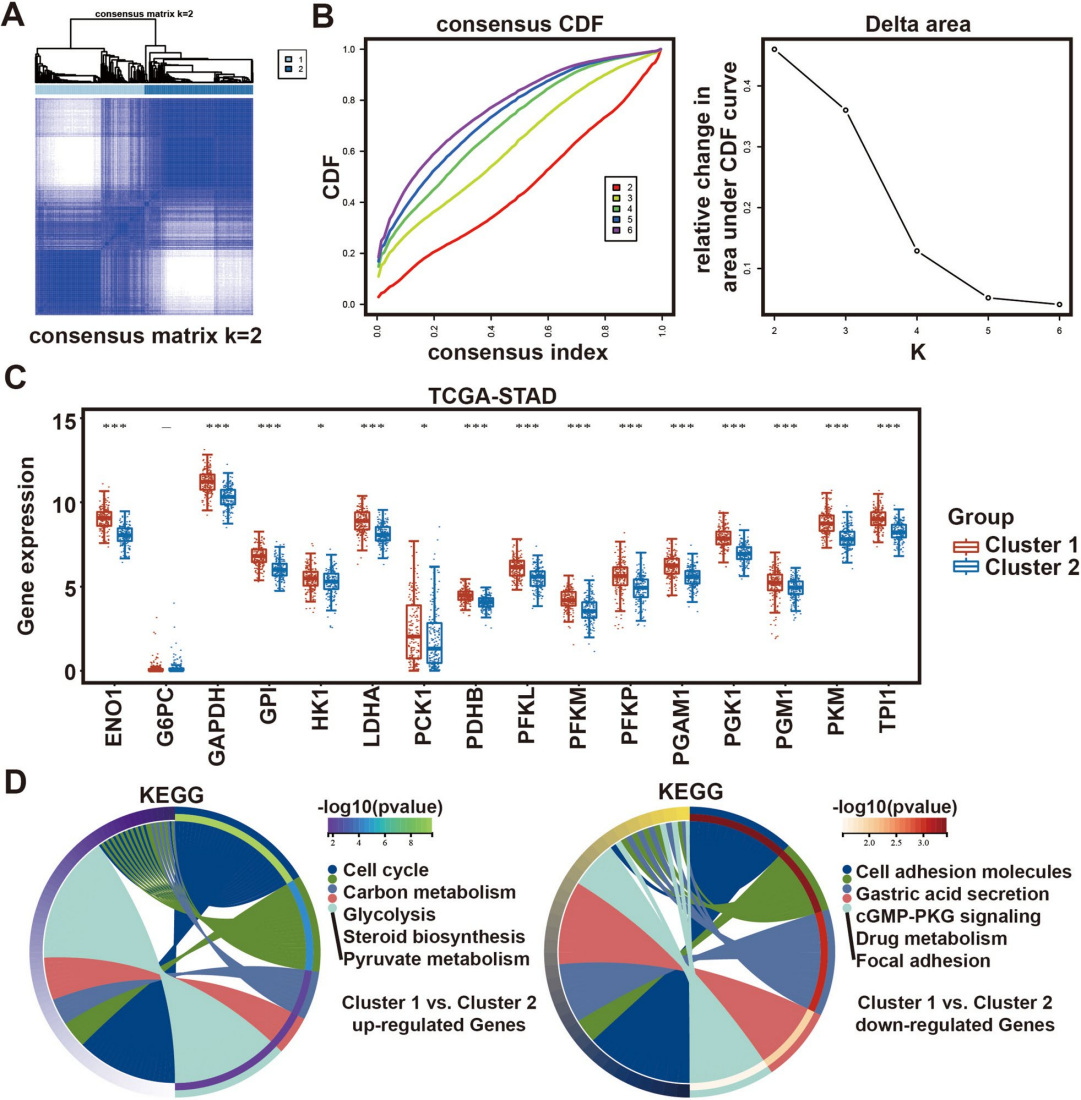
Identification of subclasses identification based on 62 glycolysis-related genes using NMF consensus clustering.** A** Consensus matrix heatmap for *k* = 2. **B** CDF value of consensus index and relative change in area under CDF curve for *k* = 2–6. **C** Differential expression of glycolytic-related genes between cluster 1 and cluster 2.** D** The circle plot of KEGG pathway enrichment analysis

The NMF cluster analysis employed a set of 62 genes linked to glycolysis. The optimal *k* value of 2 was determined using the comprehensive correlation coefficient (Fig. 1A). The TCGA-STAD samples were divided into two distinct clusters: cluster 1 (*n* = 188) and cluster 2 (*n* = 187). At *k* = 2, the consensus matrix’s heatmap exhibited clear boundaries and minimal disruption within subgroups, indicating the existence of stable clusters in the samples (Fig. 1B; Fig. S1A-D). Besides, as illustrated in Fig. 1C and Fig. S1E, in cluster 1, a significant majority of the 62 genes exhibit upregulation in comparison to cluster 2.

Figure S2A, B illustrates the volcano plot and heatmap of differentially expressed genes (DEGs) in two clusters, providing a comprehensive understanding of the glycolysis status in the samples. Cluster 1 exhibited a noteworthy upregulation of 127 genes and a significant downregulation of 58 genes (*P* < 0.05). The pathway enrichment analysis revealed a strong association between the differentially expressed genes (DEGs) and glycolysis and cell cycle, specifically carbon metabolism and pyruvate metabolism (Fig. 1D).

### Correlation analysis of TOP1MT and glycolysis in gastric cancer

**Fig. 2:**
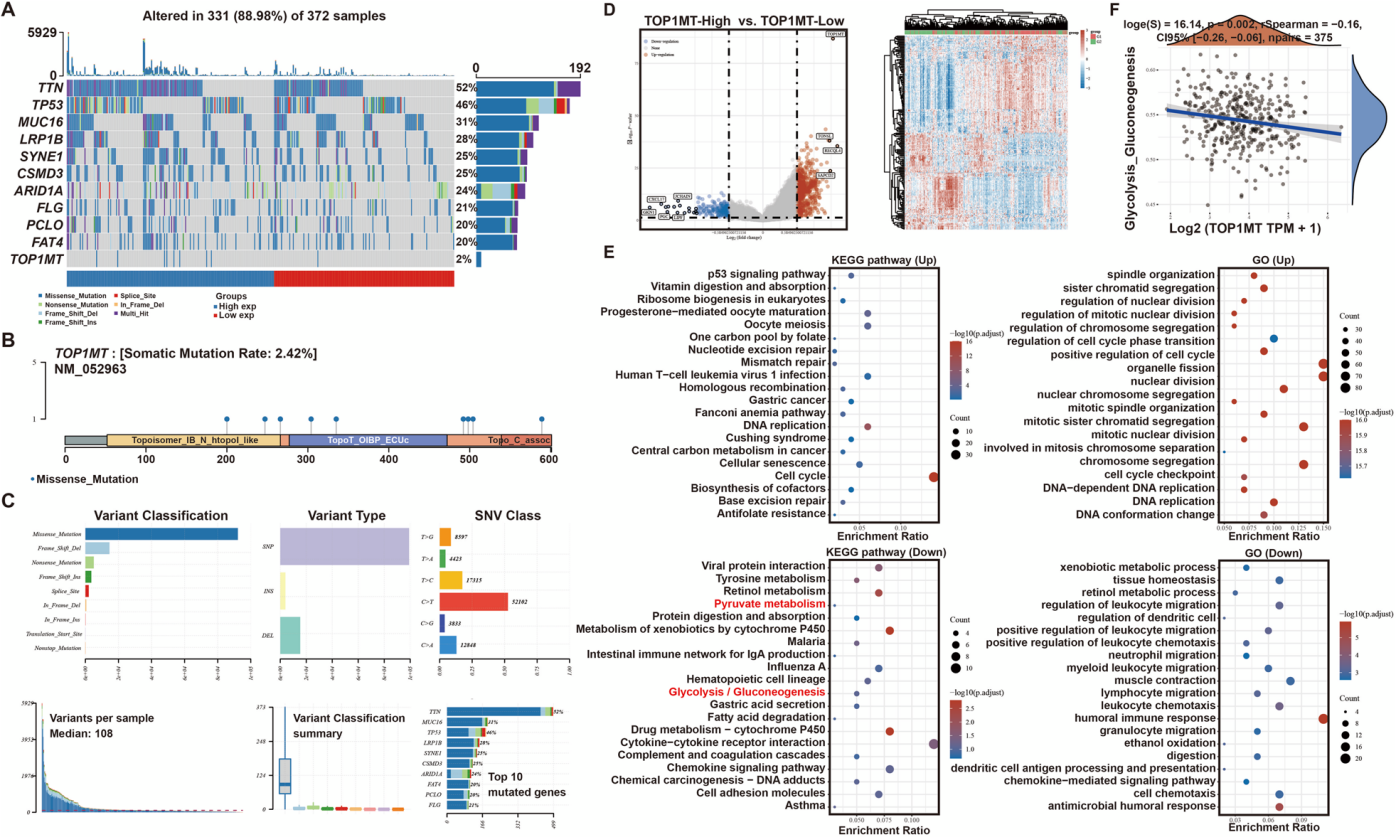
TOP1MT-related signaling pathway analysis. **A** The somatic landscape of a gastric cancer cohort. Genes are ordered by mutation frequency. **B** Lollipop charts of the mutated TOP1MT gene. **C** Cohort summary plot shows the distribution of variants according to variant classification, type and SNV class. **D** The volcano plot was constructed using the fold change values and P-adjust (TOP1MT-high vs. TOP1MT-low). **E** KEGG pathway enrichment and GO term enrichment results of differential genes. **F** The correlations between TOP1MT and glycolytic/gluconeogenesis pathway score were analyzed with Spearman

We applied the R tool to evaluate the top 10 genes and TOP1MT with mutation rates in GC patients. TTN mutations had the largest frequency in high-risk groups, while the mutation rate of TOP1MT is only 2.42% (Fig. 2A–C).

According to the expression level of TOP1MT in the samples of gastric cancer patients, they are divided into two groups, and the DEGs between the two groups are shown in Fig. 2D. Some of the genes downregulated in the TOP1MT high-expression group were involved in the process of glycolysis and gluconeogenesis (Fig. 2E). Moreover, the correlations between TOP1MT and pathway score was analyzed with Spearman, and results showed that TOP1MT was negatively correlated with glycolysis and gluconeogenesis in gastric cancer (Fig. 2F). Therefore, based on the above findings, TOP1MT was highly likely to negatively regulate the glycolysis of GC cells.

### Identification of the core modules related to TOP1MT by WGCNA

**Fig. 3:**
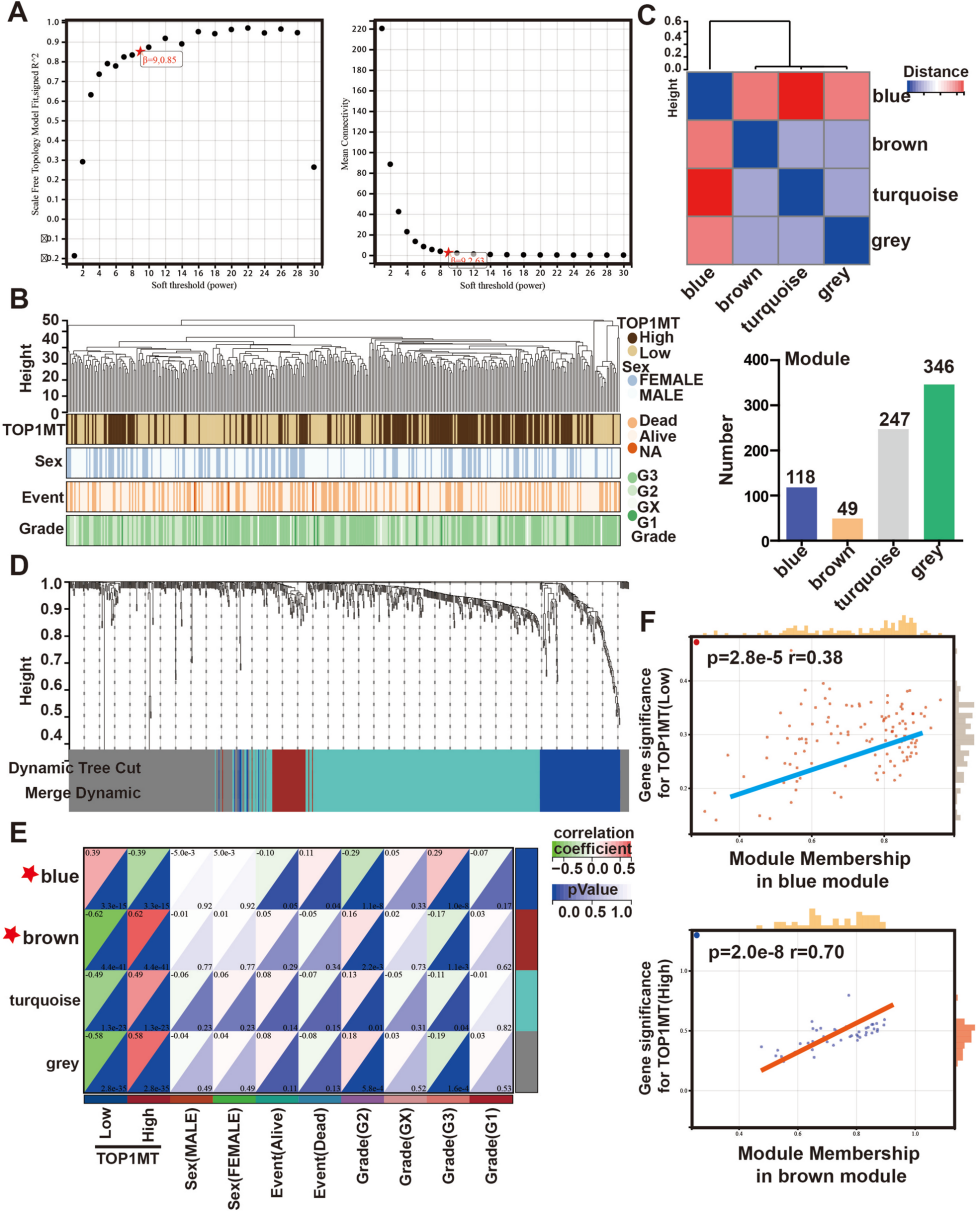
The co-expression network constructed based on the TCGA-STAD dataset.** A** The soft thresholding and correlation coefficient in the scale-free topology fitting graph. **B** Mapping of clinical trait variables and aggregation trees show the landscape of the expression of TOP1MT, sex, event, and grade of GC patients. **C** Module feature vector clustering. **D** The dynamic tree that displays different color-coded co-expression modules was constructed. **E** Correlation between module eigengenes and clinical traits. **F** The brown module was identified to have the highest positive correlation with the TOP1MT expression, while the blue modules were inversely correlated

Based on 760 DEG expression matrices and 374 clinical data networks from STAD samples, we constructed a WGCNA co-expression network. As shown in Fig. 3A, the soft threshold of *β* = 9 was identified. Four clinical variables were applied in the WGCNA (Fig. 3B): TOP1MT Expression (high or low), sex, events, and tumor grade. All DEGs with similar expression patterns clustered into the same modules, and modules showing a difference in cut height < 0.25 were merged. Four co-expression modules were yielded in this procedure, including blue, brown, turquoise, and gray. Each module had different colors and genes (Fig. 3C). These modules were significantly independent of each other, and the gene expressions in each module also showed significant independence. In addition, eigengene module values were calculated for each module, and a clustering tree is shown in Fig. 3D. The characteristic genes of the brown module were strongly positively correlated with TOP1MT-high expression, while the blue module showed opposite (Fig. 3E). Besides, in these modules, MM scores were positively correlated with GS scores (Fig. 3F). These results indicated that the genes in the blue and brown integrated modules were closely related to the expression of TOP1MT in GC. Therefore, these two modules were used to analyze the hub genes.

### Identification of final hub genes

**Fig. 4:**
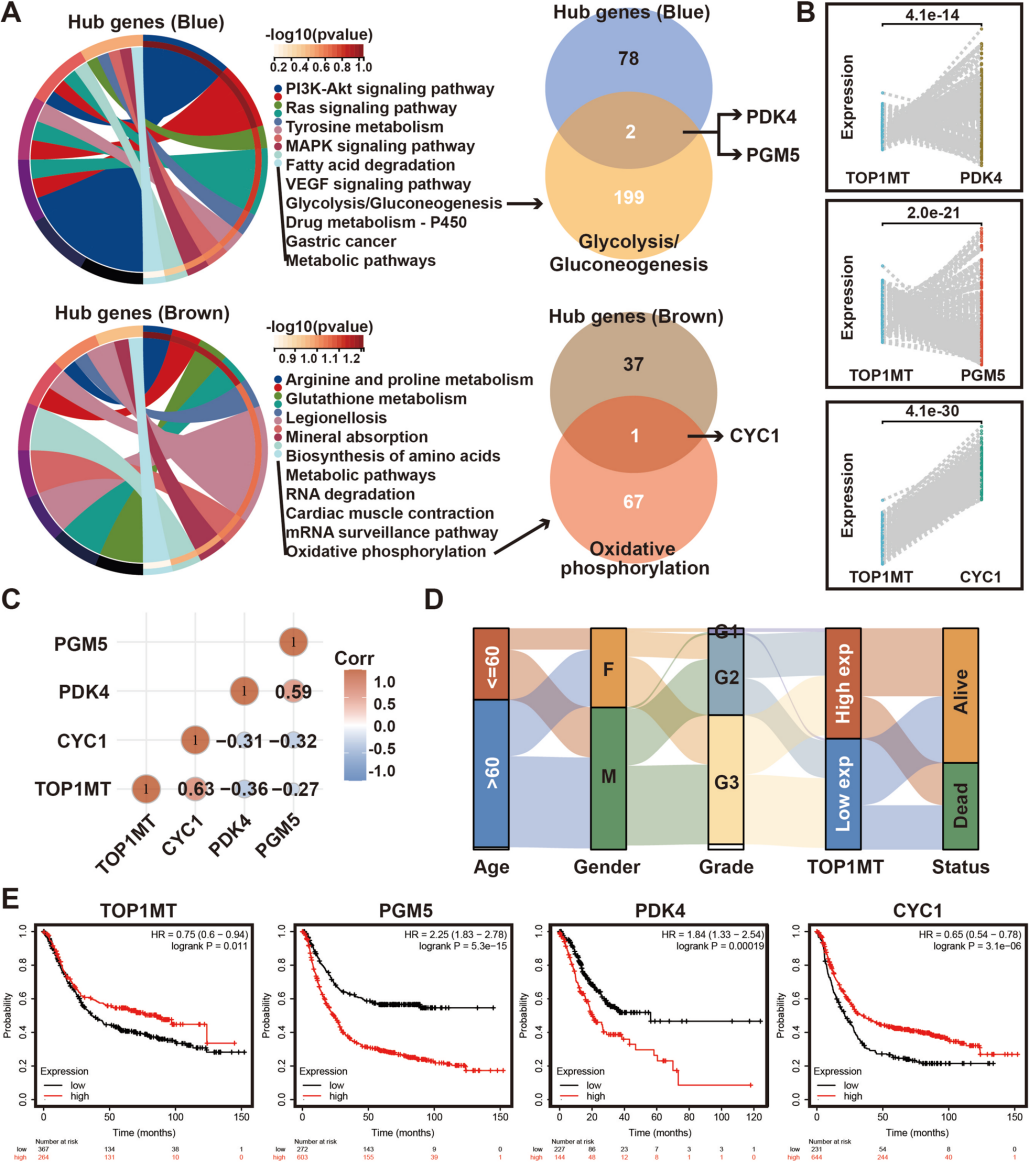
Screening and prognostic analysis of hub genes. **A** KEGG pathway enrichment analysis of blue and brown hub genes. **B** Paired visual analysis of the expression levels of TOP1MT and PDK4, PGM5, and CYC1 in GC samples. **C** The expression correlation of two genes was analyzed with Spearman. **D** Sankey energy shunt showed the distribution trend of TOP1MT expression in different clinical features. **E** Kaplan–Meier analysis of the correlation between the expression of these four genes and OS in TCGA-STAD, severally

To explore the cell signals and pathways involved in Hub genes, KEGG pathway enrichment analysis displayed that blue module genes mainly participated in cell growth signals, such as RAS signaling, and cellular glycolipid metabolism, such as glycolysis/gluconeogenesis and fatty acid degradation. The genes of the brown module were mainly involved in oxidative phosphorylation (OXPHOS) and amino acid metabolism. Subsequently, the hub genes most relevant to TOP1MT by WGCNA were compared with 199 glycolysis-related genes and 67 oxidative phosphorylation genes, respectively. Then, PDK4, PGM5, and CYC1 were found to be the overlapped genes in both results (Fig. 4A), indicating that these genes were not only highly correlated with the TOP1MT expression, but also played a key role in intracellular glucose metabolism.

Correlation analysis of mRNA levels showed that TOP1MT was positively correlated with CYC1 and negatively correlated with PDK4 and PGM5 (Fig. 4B, C), and this finding is consistent with the previous correlation analysis of TOP1MT and modules by WGCNA (Fig. 3F). We revealed that high TOP1MT expression was significantly associated with better overall survival for GC through the Sankey diagram and Kaplan–Meier analysis. Meanwhile, Kaplan–Meier analysis of other genes showed that PDK4 and PGM5 were risk factors, while CYC1 was a protective factor (Fig. 4D, E).

### PDK4 was selected as a hub gene affecting the prognosis of GC patients

**Fig. 5:**
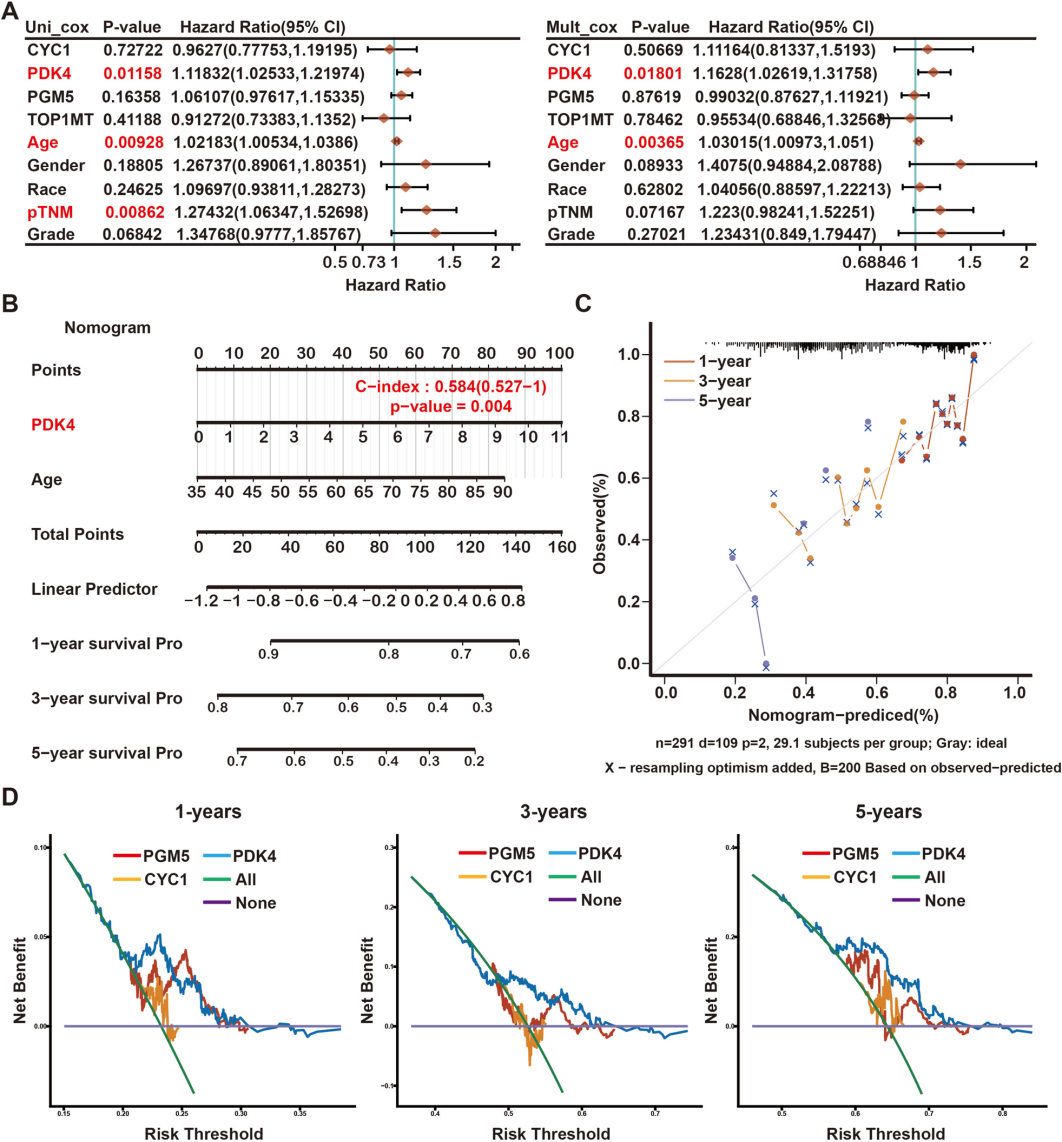
Construction of a PDK4-based prognostic model. **A** Univariate and multivariate Cox regression analyses of TOP1MT, PDK4, PGM5, and CYC1 levels with age, gender, race, pTNM stage, and grade in TCGA-STAD cohorts. HR and *p* values were displayed. **B** Nomogram by multivariate Cox regression analysis for predicting the proportion of patients with OS. **C** Plots depict the calibration of the model in terms of agreement between predicted and observed OS. **D** DCA of candidate mRNAs (PDK4, PGM5, and CYC1) for predicting survival status

The expression of PDK4 was the only variable that showed statistical significance in the univariate Cox regression analysis. In the multivariate Cox proportional hazards regression model, PDK4 was assessed as a distinct prognostic biomarker using the TCGA data (HR = 1.16, 95% confidence interval (CI) = 1.03–1.32, *p* = 0.018; Fig. 5A).

To improve the prognostic prediction of patients with gastric cancer (GC) in a clinical setting, a prognostic nomogram was developed by integrating two mortality indicators, namely PDK expression and patient age, in a multivariate Cox regression model. The accuracy and validity of this model were subsequently evaluated and validated using TCGA. The nomogram developed in this study was utilized to calculate a score for predicting the overall survival (OS) of individual patients with gastric cancer at 1, 3, and 5 years (Fig. 5B). The calibration plot (Fig. 5C) closely resembled an ideal model, demonstrating the nomogram’s accuracy in predicting patient overall survival (OS). In terms of predicting overall survival (OS), the decision curve analysis (DCA) demonstrated that PDK4 expression had the highest accuracy among the analyzed factors (Fig. 5D). Given the importance of PDK4 in gastric cancer (GC) and its correlation with TOP1MT, this forthcoming study aims to examine the relationship between TOP1MT and PDK4 in pathology. Additionally, it will investigate the potential regulatory role of TOP1MT on PDK4.

### TOP1MT was linked with the clinicopathological characteristics of GC

**Fig. 6:**
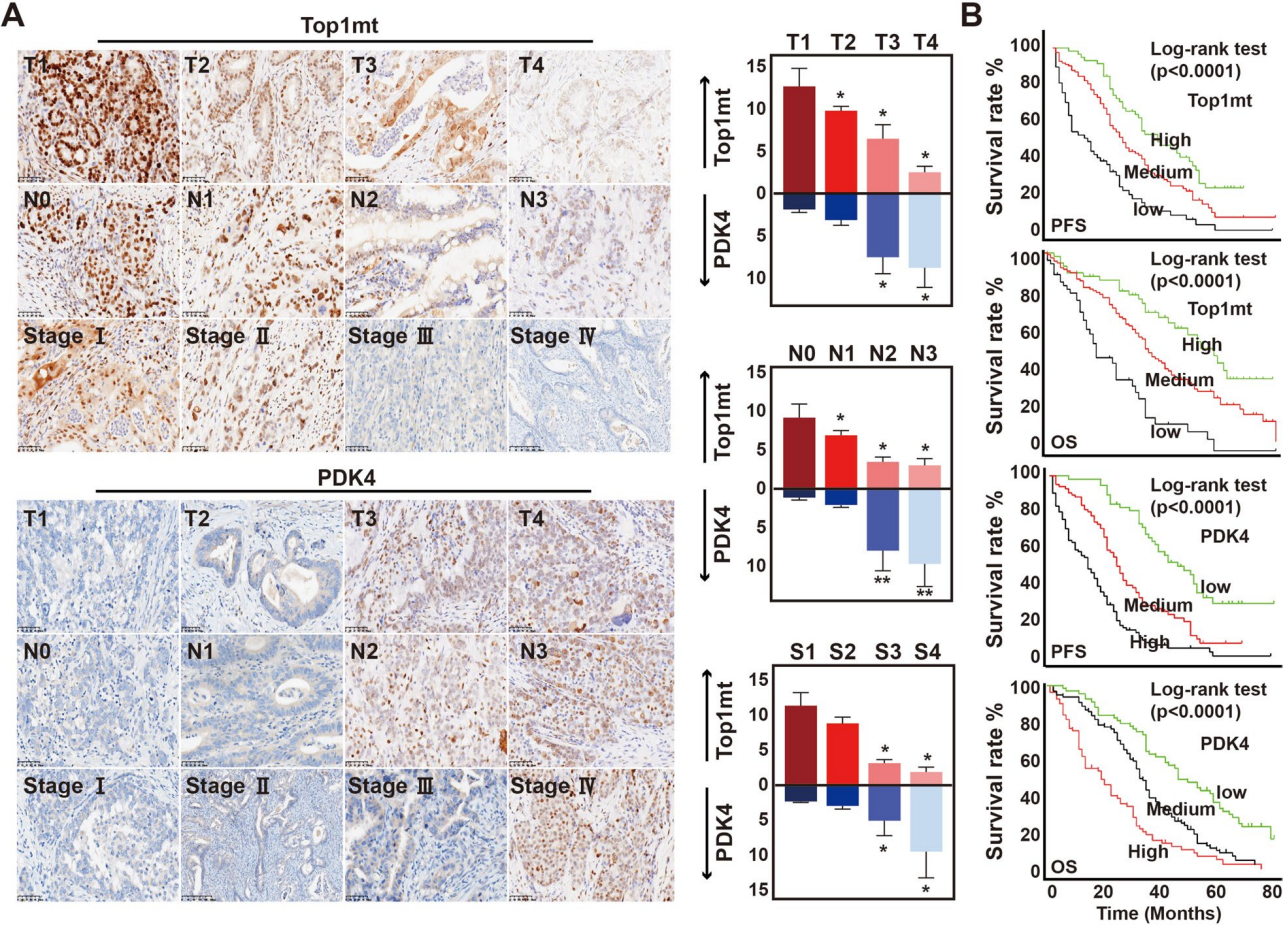
TOP1MT expression in GC patients and its correlation with the clinicopathological characteristics.** A** Representative images of immunohistochemical staining of TOP1MT and PDK4 expression in each group related to TNM stage (magnification, 200 ×). **B** Kaplan–Meier survival curves (PFS and OS) of TOP1MT and PDK4 in patients with GC. *P* values * < 0.05

To further examine the correlation between TOP1MT and PDK4 in clinical samples, we analyzed tumor samples from 250 patients diagnosed with gastric cancer. Subsequently, we conducted immunohistochemical staining to investigate the correlations between TOP1MT/PDK4 expression and clinicopathological characteristics in patients with gastric cancer. During the follow-up period, patients with GC at more advanced stages exhibited a significant decline in TOP1MT expression, as evidenced by immunohistochemical staining using TOP1MT antibodies. In this cohort of 250 GC patients, the immunohistochemical analysis of PDK4 in successive sections showed a significant increase in PDK4 expression in patients with more advanced stages of the disease (Fig. 6A).

GC patients exhibiting low TOP1MT expression experienced shorter progression-free survival (PFS) rates in stage I–III cancer, as well as lower overall survival rates in stage IV cancer, in comparison to patients with high TOP1MT. PDK4, on the other hand, showed the opposite trend (Fig. 6B). Through the analysis of consecutive GC sections, a noteworthy correlation between TOP1MT expression and PDK4 expression was identified.

### Silencing of TOP1MT induced GC cell migration by targeting PDK4

**Fig. 7:**
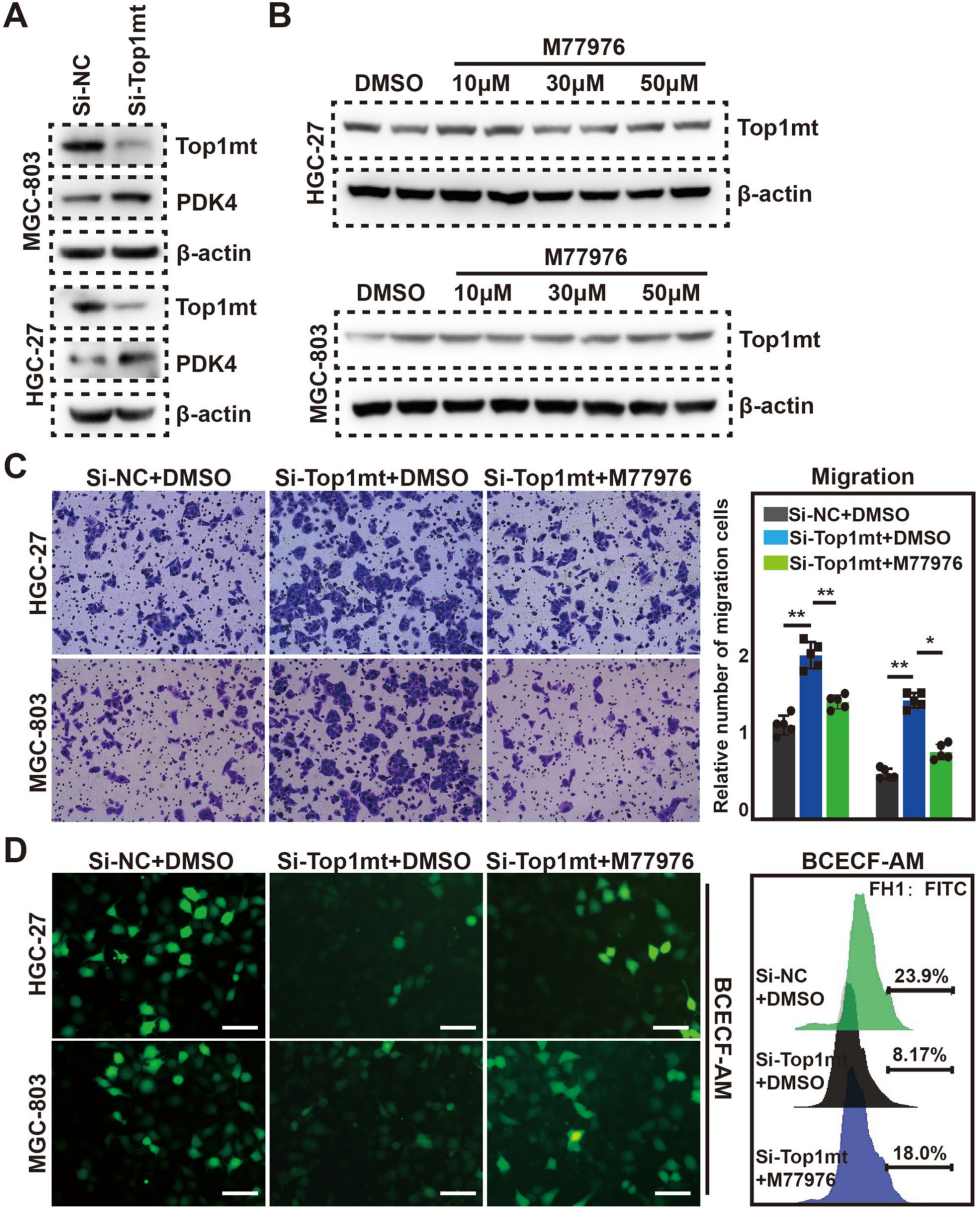
PDK4 was required for the upregulation of migration and lactate production in GC cells induced by TOP1MT silencing. **A** Western blot analysis for the detection of PDK4 expression in MGC-803 and HGC-27 cells. **B** The effects of M77976 on TOP1MT expression were analyzed by western blot. **C** Cell migration was assessed by transwell migration assays. **D** Representative experiments showing pH determination (BCECF-AM) after 48 h of culture in different groups. *P* values * < 0.05 and ** < 0.01

To explore the effects of low TOP1MT expression on GC cells, we performed functional experiments in HGC-27 and MGC-803 cells. Cells were transfected with siRNA targeting TOP1MT. Therefore, we chose the siRNA (si-TOP1MT1) used in the previous study to knock down TOP1MT [18]. Results displayed that PDK4 protein expression was significantly increased in HGC-27 and MGC-803 cells with TOP1MT knockdown (Fig. 7A). This finding demonstrated that PDK4 expression was enhanced in GC cells with TOP1MT silencing. Meanwhile, PDK4 inhibitor M77976 had no significant effect on the expression level of TOP1MT (Fig. 7B).

The numbers of migrating cells were significantly higher for siRNA-transfected HGC-27 and MGC-803 cells, and M77976 could partially restore the inhibition of cell migration caused by TOP1MT knockdown (Fig. 7C). Besides, the results of pH fluorescence probe labeling showed that TOP1MT silencing significantly decreased the pH value in HGC-27 and MGC-803 cells, indicating increased lactate content (Fig. 7D). These data indicated that glycolysis was enhanced in GC cells with TOP1MT silencing.

### Silencing of TOP1MT increased ECAR and decreased OCR in GC cells

**Fig. 8:**
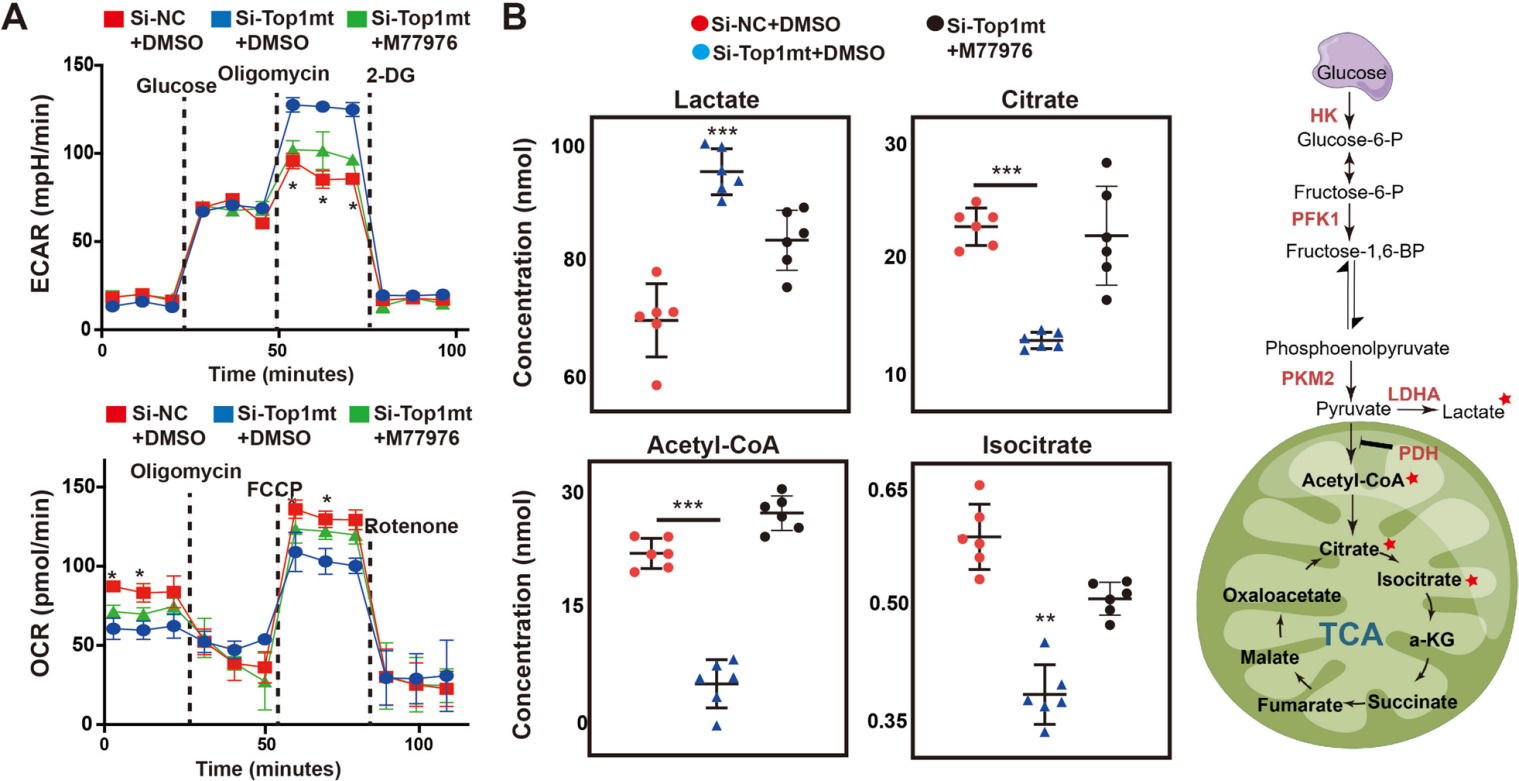
Intracellular metabolism level was detected after TOP1MT knockdown.** A** Oxygen consumption rate (OCR) and extracellular acidification rate (ECAR) measured by Seahorse XF296 Extracellular Flux Analyzer. **B** Mass spectrometry analysis of 4 main intermediate metabolites during glycolysis and TCA cycles. *P* values * < 0.05, ** < 0.01, and *** < 0.001

In the previous experiment, we assessed the effect of TOP1MT on glycolysis in GC cells via detecting intracellular pH value. Next, in order to more directly observe the regulatory effect of TOP1MT on glucose metabolism, the sea-horse assays were used to evaluate the influence of TOP1MT silencing on acid production and oxygen consumption of GC cells. These two indicators represented the glycolysis and oxidative phosphorylation capacity of cells, respectively. As shown in Fig. 8A, TOP1MT knockdown significantly increased acid production and decreased oxygen consumption in HGC-27 and MGC-803 cells, suggesting an increase in glycolysis and a decrease in oxidative phosphorylation. Similarly, this metabolic change can be partially reversed by M77976.

The mass spectrometry analysis of energy metabolites also showed lactate, an important intermediate metabolite of glycolysis, increased significantly, while the metabolites of the TCA cycle, such as citrate, acetyl-CoA, and isocitrate decreased significantly in the case of TOP1MT knocking down, and the inhibitor M77976 can also restore the levels of these metabolites (Fig. 8B).

## Discussion

The presence of TOP1MT, a type IB topoisomerase, has been identified in humans, with a specific role in mitochondria [19–22]. The TOP1MT enzyme facilitates the relaxation of the supercoiled structure of mitochondrial DNA (mtDNA) via the formation of temporary cutting complexes, which induce the rotation of damaged DNA strands around undamaged strands [23, 24]. The genetic information of mitochondria is tightly packed in mtDNA, and both strands are actively transcribed. There is a single noncoding regulatory region that contains the promoters for mtDNA transcription. Bidirectional transcription of mtDNA results in the accumulation of negative supercoils behind the RNA polymerases due to its circular nature. To address this, TOP1mt activity is required, which is similar to the known role of nuclear TOP1 in transcription [25, 26]. In addition to regulating mtDNA, TOP1MT also interacts with small mitochondrial subunits to influence mitochondrial translation [27]. Therefore, Inhibitors targeting TOP1MT could be a viable strategy to target mitochondrial DNA due to their involvement in mitochondrial protein synthesis and their heightened expression in different tumor types.

Different tissues have different metabolic profiles because of their unique functions. Moreover, it is imperative to adjust energy requirements based on physiological or pathological conditions [28]. Experiments demonstrate that the absence of Top1mt inhibits mtDNA synthesis, providing insight into the molecular mechanisms underlying Top1mt’s role in mtDNA adaptation. During cell growth, an increase in mitochondrial mass and mtDNA is necessary to adequately supply the offspring cells [29]. The presence of intact mtDNA is essential for the synthesis of enzymatic components in the respiratory chain of mitochondria, which in turn allows for ATP production.

**Fig. 9:**
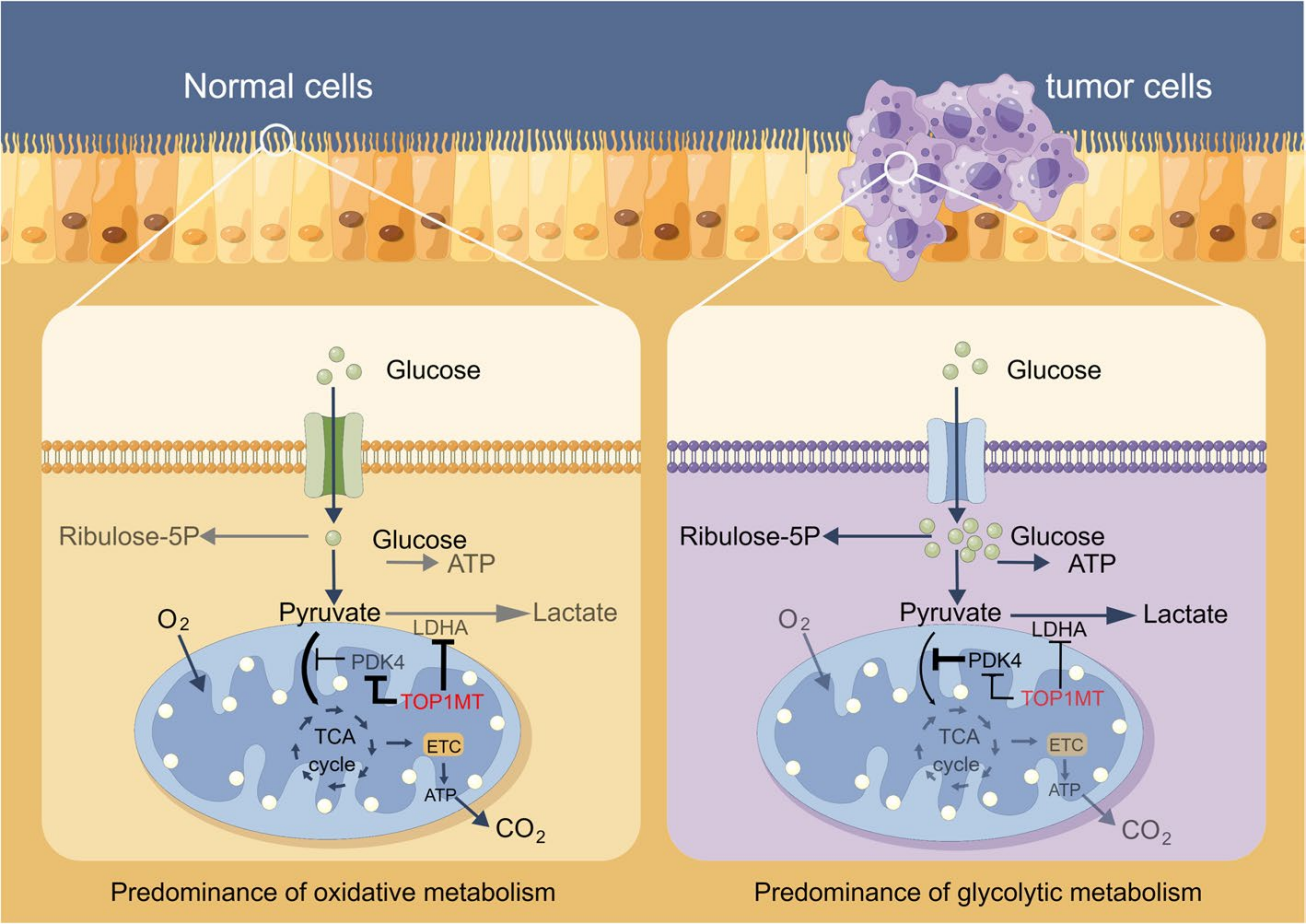
Schematic diagram of TOP1MT regulating glycolytic metabolic pathway

This study demonstrates the crucial role of TOP1MT in promoting gastric cancer progression by regulating aerobic glycolysis. To evaluate the influence of TOP1MT on GC migration and its associated metabolic mechanism, we conducted experiments on two GC cell lines and analyzed clinical data from a cohort of 250 GC patients. The deficiency of TOP1MT significantly increases glucose consumption and lactate production and promotes metastasis of GC cells, indicating an upregulation of glycolysis. Besides, the inhibition of TOP1MT resulted in a significant increase in LDHA mRNA and protein expression, which is known to play a critical role in glycolysis [14]. Our investigation reveals the interesting finding that TOP1mt not only governs LDHA but also modulates the expression of PDK4 (Fig. 9). Generally, PDK4 and LDHA collaborate to enhance the glycolysis of cancer cells [30–33]. Therefore, in conjunction with our ongoing research, TOP1MT has the potential to play a crucial role in regulating tumor metabolism and could contribute to the development of novel treatment strategies for gastric cancer.

The Warburg effect, also referred to as aerobic glycolysis, was observed to be significantly elevated in GC cells as a result of TOP1MT deficiency. According to research, cancer cells primarily rely on the Warburg effect for glucose metabolism to generate energy and support biosynthesis, even in the presence of adequate oxygen [34]. Interestingly, only 5% of glucose is metabolized through the OXPHOS pathway in the mitochondria of cancer cells [35]. The deficiency of TOP1MT in GC cells may further inhibit OXPHOS and upregulate the Warburg effect. Our experiment confirmed that inhibiting TOP1MT significantly reduces the oxygen consumption (OCR) of GC cells and simultaneously increases the production of lactic acid (ECAR) in cells. Meanwhile, the metabolic changes induced by TOP1MT knockdown can be partially restored by PDK4 inhibitor M77976. The study’s findings indicate that the absence of TOP1MT plays a role in the proliferation of cancer cells, particularly through the Warburg effect. These results emphasize the potential importance of PDK4 in facilitating this process.

The accelerated growth rate and invasive capabilities of cancer cells result in increased oxygen consumption, which meets the various biosynthetic needs of these cells. Rapid tumor growth consistently results in intratumoral hypoxia. The oxygen deficiency in this environment induces the activation of HIF-1a, subsequently initiating the activation of its target genes [36, 37]. HIF-1 governs LDHA transcription. The activation of HIF-1a leads to the upregulation of LDHA, which promotes the conversion of pyruvate to lactate and decreases the influx of pyruvate into mitochondria. PDK can simultaneously inhibit pyruvate dehydrogenase complex (PDC), disrupt TCA cycle, and promote the conversion of pyruvate to lactate. This procedure has the potential to enhance glycolysis while impairing mitochondrial oxidative phosphorylation (OXPHOS). The heightened Warburg phenomenon leads to increased lactate production, which in turn promotes the formation of an acidic microenvironment. This finding suggests a potential mechanism for increased metastasis and invasion in tumor cells.

In conclusion, this report primarily examined the role of TOP1MT in gastric cancer. We have identified a new mechanism that facilitates the dissemination of GC in the absence of TOP1MT. Furthermore, our findings demonstrate the substantial role of TOP1MT in the functioning of cancer cells. Therefore, upregulating TOP1MT expression and employing PDK4 inhibitors may impede the dissemination of GC.

### Supplementary Information

**Additional file 1. **Supplementary method. **Figure S1.** Identification of subclasses identification based on 62 glycolysis-related genes using NMF consensus clustering in TCGA-STAD. (A) Consensus matrix legend; (B) The tracking plot for k = 2–6; (C) The heat-map for K = 2; (D) Consensus matrix heat-map for k = 3–6; (E) The differential expression of glycolytic-related genes between cluster 1 and cluster 2. **Figure S2.** The results of KEGG and GO enrichment analysis based on DEGs between cluster 1 and cluster 2 in TCGA-STAD. (A) Volcanic map; (B) Heat-map.

## Data Availability

The data generated in this study are available upon request from the corresponding author.

## References

[1] López MJ, Carbajal J, Alfaro AL, Saravia LG, Zanabria D, Araujo JM (2023). Characteristics of gastric cancer around the world. Crit Rev Oncol Hematol.

[2] Ugai T, Sasamoto N, Lee HY, Ando M, Song M, Tamimi RM (2022). Is early-onset cancer an emerging global epidemic? Current evidence and future implications. Nat Rev Clin Oncol.

[3] Smyth EC, Nilsson M, Grabsch HI, van Grieken NC, Lordick F (2020). Gastric cancer. Lancet.

[4] Decourtye-Espiard L, Guilford P (2023). Hereditary diffuse gastric cancer. Gastroenterology.

[5] Zhao M, Wei F, Sun G, Wen Y, Xiang J, Su F (2022). Natural compounds targeting glycolysis as promising therapeutics for gastric cancer: a review. Front Pharmacol.

[6] Wang Y, Luo W, Li Y (2023). [68Ga]Ga-FAPI-04 PET MRI/CT in the evaluation of gastric carcinomas compared with [18F]-FDG PET MRI/CT: a meta-analysis. Eur J Med Res.

[7] Wang Y, Zheng K, Huang Y, Xiong H, Su J, Chen R (2021). PARP inhibitors in gastric cancer: beacon of hope. J Exp Clin Cancer Res.

[8] Yuan LW, Yamashita H, Seto Y (2016). Glucose metabolism in gastric cancer: The cutting-edge. World J Gastroenterol.

[9] Wang X, Xu B, Du J, Xia J, Lei G, Zhou C (2022). Characterization of pyruvate metabolism and citric acid cycle patterns predicts response to immunotherapeutic and ferroptosis in gastric cancer. Cancer Cell Int.

[10] Menger KE, Chapman J, Díaz-Maldonado H, Khazeem MM, Deen D, Erdinc D (2022). Two type I topoisomerases maintain DNA topology in human mitochondria. Nucleic Acids Res.

[11] Al Khatib I, Deng J, Symes A, Kerr M, Zhang H, Huang SN (2022). Functional characterization of two variants of mitochondrial topoisomerase TOP1MT that impact regulation of the mitochondrial genome. J Biol Chem.

[12] Baechler SA, Dalla Rosa I, Spinazzola A, Pommier Y (2019). Beyond the unwinding: role of TOP1MT in mitochondrial translation. Cell Cycle.

[13] Baechler SA, Factor VM, Dalla Rosa I, Ravji A, Becker D, Khiati S (2019). The mitochondrial type IB topoisomerase drives mitochondrial translation and carcinogenesis. Nat Commun.

[14] Wang H, Zhou R, Sun L, Xia J, Yang X, Pan C (2017). TOP1MT deficiency promotes GC invasion and migration via the enhancements of LDHA expression and aerobic glycolysis. Endocr Relat Cancer.

[15] Wang L, Wu Y, Lin L, Liu P, Huang H, Liao W (2013). Metastasis-associated in colon cancer-1 upregulation predicts a poor prognosis of gastric cancer, and promotes tumor cell proliferation and invasion. Int J Cancer.

[16] Kukimoto-Niino M, Tokmakov A, Terada T, Ohbayashi N, Fujimoto T, Gomi S (2011). Inhibitor- bound structures of human pyruvate dehydrogenase kinase 4. Acta Crystallogr D Biol Crystallogr.

[17] Ramesh K, Hu MY, Melzner F, Bleich M, Himmerkus N (2020). Intracellular pH regulation in mantle epithelial cells of the Pacific oyster Crassostrea gigas. J Comp Physiol B.

[18] Laudette M, Lindbom M, Arif M, Cinato M, Ruiz M, Doran S (2023). Cardiomyocyte-specific PCSK9 deficiency compromises mitochondrial bioenergetics and heart function. Cardiovasc Res.

[19] Zhang H, Meng LH, Pommier Y (2007). Mitochondrial topoisomerases and alternative splicing of the human TOP1mt gene. Biochimie.

[20] Zhang H, Zhang YW, Yasukawa T, Dalla Rosa I, Khiati S, Pommier Y (2014). Increased negative supercoiling of mtDNA in TOP1mt knockout mice and presence of topoisomerases IIα and IIβ in vertebrate mitochondria. Nucleic Acids Res.

[21] Douarre C, Sourbier C, Dalla Rosa I, Brata Das B, Redon CE, Zhang H (2012). Mitochondrial topoisomerase I is critical for mitochondrial integrity and cellular energy metabolism. PLoS ONE.

[22] Zhang H, Pommier Y (2008). Mitochondrial topoisomerase I sites in the regulatory D-loop region of mitochondrial DNA. Biochemistry.

[23] Dalla Rosa I, Huang SY, Agama K, Khiati S, Zhang H, Pommier Y (2014). Mapping topoisomerase sites in mitochondrial DNA with a poisonous mitochondrial topoisomerase I (Top1mt). J Biol Chem.

[24] Zhang H, Barceló JM, Lee B, Kohlhagen G, Zimonjic DB, Popescu NC (2001). Human mitochondrial topoisomerase I. Proc Natl Acad Sci U S A.

[25] Ballot C, Kluza J, Lancel S, Martoriati A, Hassoun SM, Mortier L (2010). Inhibition of mitochondrial respiration mediates apoptosis induced by the anti-tumoral alkaloid lamellarin D. Apoptosis.

[26] Khiati S, Seol Y, Agama K, Dalla Rosa I, Agrawal S, Fesen K (2014). Poisoning of mitochondrial topoisomerase I by lamellarin D. Mol Pharmacol.

[27] Kolesar JE, Wang CY, Taguchi YV, Chou SH (2013). Two-dimensional intact mitochondrial DNA agarose electrophoresis reveals the structural complexity of the mammalian mitochondrial genome. Nucleic Acids Res.

[28] Lu Z, Wu S, Yan C, Chen J, Li Y (2021). Clinical value of energy spectrum curves of dual-energy computer tomography may help to predict pathological grading of gastric adenocarcinoma. Transl Cancer Res.

[29] Popov LD (2020). Mitochondrial biogenesis: An update. J Cell Mol Med.

[30] Liu B, Zhang Y, Suo J (2021). Increased expression of PDK4 was displayed in gastric cancer and exhibited an association with glucose metabolism. Front Genet.

[31] Miao Y, Li Q, Sun G, Wang L, Zhang D, Xu H, Xu Z (2020). MiR-5683 suppresses glycolysis and proliferation through targeting pyruvate dehydrogenase kinase 4 in gastric cancer. Cancer Med.

[32] Jiang W, Zhou F, Li N, Li Q, Wang L (2015). FOXM1-LDHA signaling promoted gastric cancer glycolytic phenotype and progression. Int J Clin Exp Pathol.

[33] Wang XH, Jiang ZH, Yang HM, Zhang Y, Xu LH (2021). Hypoxia-induced FOXO4/LDHA axis modulates gastric cancer cell glycolysis and progression. Clin Transl Med.

[34] Schwartz L, Supuran CT, Alfarouk KO (2017). The Warburg effect and the hallmarks of cancer. Anticancer Agents Med Chem.

[35] Greene J, Segaran A, Lord S (2022). Targeting OXPHOS and the electron transport chain in cancer; molecular and therapeutic implications. Semin Cancer Biol.

[36] Jing X, Yang F, Shao C, Wei K, Xie M, Shen H, Shu Y (2019). Role of hypoxia in cancer therapy by regulating the tumor microenvironment. Mol Cancer.

[37] Le A, Cooper CR, Gouw AM, Dinavahi R, Maitra A, Deck LM (2010). Inhibition of lactate dehydrogenase A induces oxidative stress and inhibits tumor progression. Proc Natl Acad Sci U S A.

