# Supplementary material for: Deficiency of TOP1MT enhances glycolysis through the stimulation of PDK4 expression in gastric cancer
Source: Cancer Metab. 2024 Jan 10;12:2. doi: 10.1186/s40170-024-00330-w (PMC10777619; doi:10.1186/s40170-024-00330-w)
Supplement: Supplementary file 1 — Additional file 1. Supplementary method. Figure S1. Identification of subclasses identification based on 62 glycolysis-related genes using NMF consensus clustering in TCGA-STAD. (A) Consensus matrix legend; (B) The tracking plot for k = 2–6; (C) The heat-map for K = 2; (D) Consensus matrix heat-map for k = 3–6; (E) The differential expression of glycolytic-related genes between cluster 1 and cluster 2. Figure S2. The results of KEGG and GO enrichment analysis based on DEGs between cluster 1 and cluster 2 in TCGA-STAD. (A) Volcanic map; (B) Heat-map. [file 40170_2024_330_MOESM1_ESM.docx]

**Supplementary materials**

**Supplementary method**

1. ***Construction of Risk Score Model by Multivariate Cox***

Univariate and multivariate cox regression analysis were performed to identify the proper terms to build the nomogram. The forest was used to show the P value, HR and 95% CI of each variable through ‘forestplot’ R package. In addition, univariate and multivariate Cox regression analyses were used to explore the prognostic efficiency of the risk models and other clinicopathological features.

1. ***Construction and Validation of a Predictive Nomogram***

A nomogram can be performed to predict the prognosis of various cancers. In the TCGA datasets, four glycolysis-related genes identified by multivariate Cox regression were included to build a nomogram using the ‘rms’ package in R to investigate the 1-, 3- and 5-year survival rate of GC patients. To assess the discrimination and accuracy of the nomogram, then we calculated the concordance index (C-index) and plotted a calibration curve.

1. ***DCA (Decision Curve Analysis)***

RNA-sequencing expression profiles and corresponding clinical information for STAD were downloaded from the TCGA dataset. Normalizing the data log2 (TPM+1), keeping samples with clinical information at the same time. We used decision curve analysis R package-ggDCA to construct 3 diagnostic models. All the above analysis methods and R packages were performed using R software version v4.1.3. P < 0.05 was considered statistically significant.

1. ***Mutation of TOP1mt in gastric cancer***

RNA-seq expression profiles and genetic mutation information for STAD were downloaded from TCGA. The data of mutations were downloaded and visualized using the maftools package in R software. Genes with higher mutational frequency detected of STAD patient in histogram was showed.

1. ***WGCNA analysis***

The WGCNA R package was used to assess the relative importance of TOP1mt and their module membership. First, the Pearson's correlation matrices and average linkage method were both performed for all pair-wise Genes, then, a weighted adjacency matrix was constructed using a power function A_mn=|C_mn|^β (C_mn = Pearson's correlation between Gene_m and Gene_n; _mn= adjacency between Gene m and Gene n). β was a soft-thresholding parameter that could emphasize strong correlations between Genes and penalize weak correlations. After choosing the power of 4, the adjacency was transformed into a topological overlap matrix (TOM), which could measure the network connectivity of a Gene defined as the sum of its adjacency with all other Genes for network Gene ration, and the corresponding dissimilarity (1TOM) was calculated. To classify Genes with similar expression profiles into Gene modules, average linkage hierarchical clustering was conducted according to the TOM-based dissimilarity measure with a minimum size (Gene group) of 30 for the Genes dendrogram. The analysis of WGCNA was completed on the online platform of SangerBox 3.0.

1. ***Mass spectrometry***

The mass spectrometry detection is completed by the APTBIO company (Shanghai Zhongke New Life Biotechnology Co., LTD.). The specific process is as follows:

- 1. *Metabolite extraction:*

Samples were taken out at -80°C, and 0.5ml methanol acetonitrile solution (2:2:1, v/v) was added, 10ul (10mMol/L SUCCINIC ACID-D6 internal standard) was added, the vortex was 60s, low temperature ultrasound was performed for 30min twice, and the protein was precipitated at -20°C for 1h, with 14000rcf. Centrifuge at 4°C for 20min, freeze-dry the supernatant, and store the sample at -80°C.

- 1. *Chromatography-mass spectrometry detection conditions:*

The samples were separated by Agilent 1290 Infinity LC ultra-high performance liquid chromatography system. Sample placement In 4°C automatic injector, the column temperature was 35°C, the mobile phase A: 50mM ammonium acetate aqueous solution +1.2% ammonium hydroxide, the mobile phase B: 1% acetyl acetone acetonitrile solution, the flow rate was 300μL /min, and the sample size was 2μL. A 5500 QTRAP mass spectrometer (SCIEX) was used for mass spectrometry in negative ion mode.

- 1. *Data analysis:*

The chromatographic peak area and retention time were extracted by Multiquant 3.0.2 software. The retention time was corrected by the standard of the target substance, and the metabolite identification was carried out.

**Supplementary Figures and Figure legends**

**
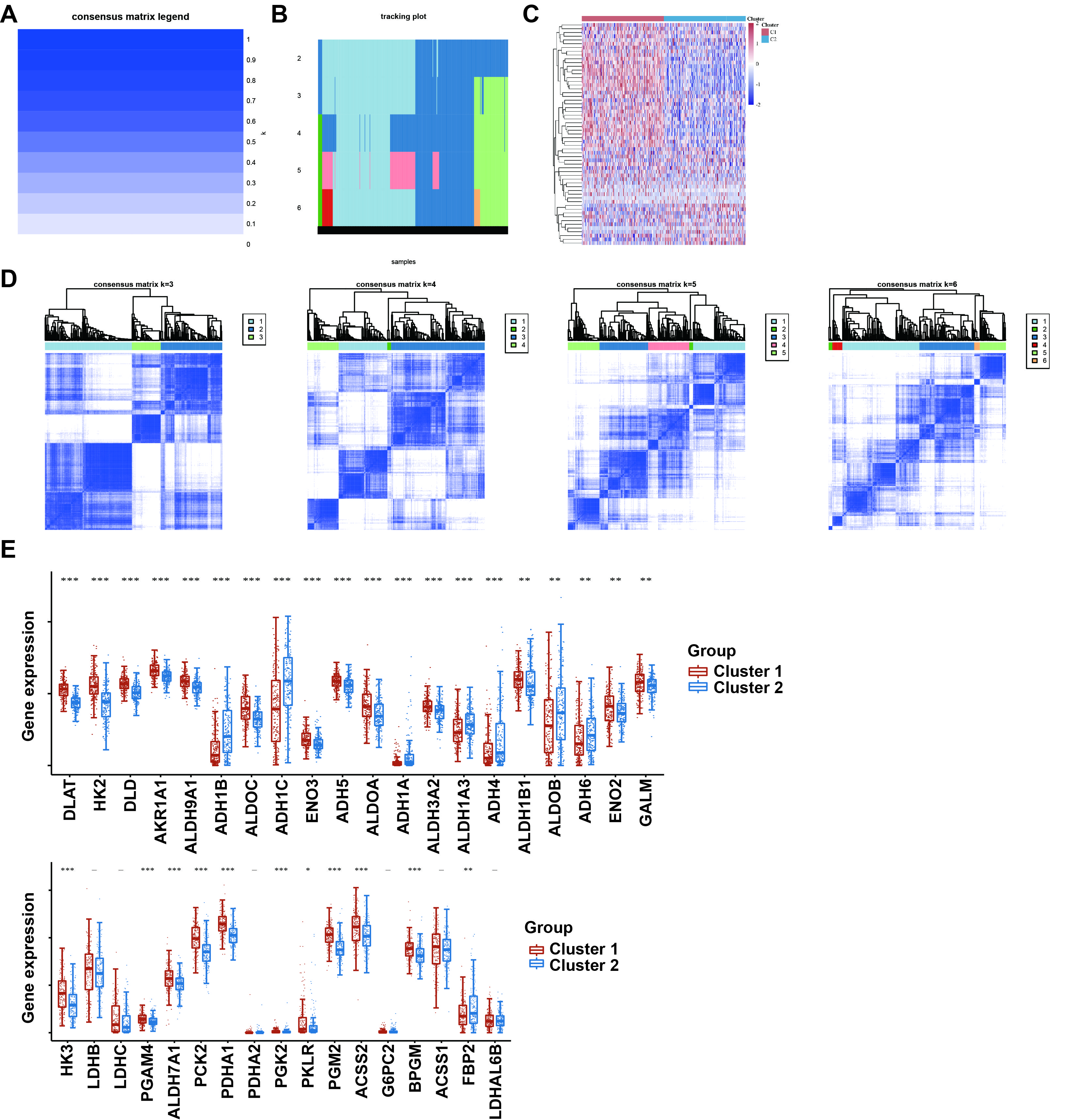
**

**Figure S1.** Identification of subclasses identification based on 62 glycolysis-related genes using NMF consensus clustering in TCGA-STAD. **(A)** Consensus matrix legend; **(B)** The tracking plot for k =2–6; **(C)** The heat-map for K = 2; **(D)** Consensus matrix heat-map for k = 3-6; **(E)** The differential expression of glycolytic-related genes between cluster 1 and cluster 2.

**
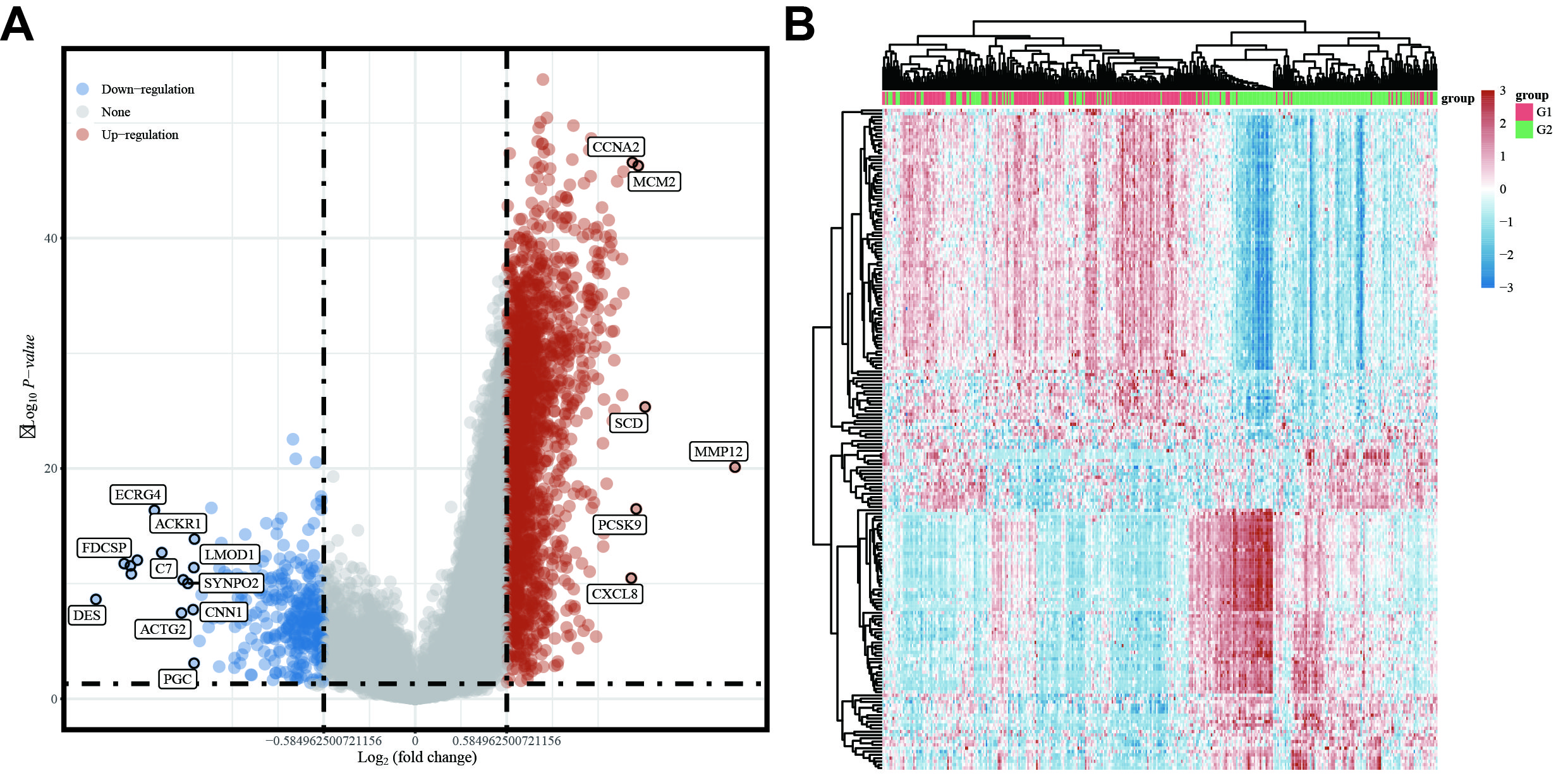
**

**Figure S2.** The results of KEGG and GO enrichment analysis based on DEGs between cluster 1 and cluster 2 in TCGA-STAD. **(A)** Volcanic map; **(B)** Heat-map.

**Short interfering RNA sequence (si-TOP1MT) targeting TOP1MT**

| Si-TOP1MT 5’ to 3’ : CCAACACGUGGUGGAAUUUTT  3’ to 5’ : AAAUUCCACCACGUGUUGGTT |
| --- |
